# Challenges in Assessing Outcomes among Infants of Pregnant HIV-Positive Women Receiving ART in Uganda

**DOI:** 10.1155/2017/3202737

**Published:** 2017-12-07

**Authors:** Barbara Castelnuovo, Frank Mubiru, Ivan Kalule, Shadia Nakalema, Agnes Kiragga

**Affiliations:** Infectious Diseases Institute, Makerere University, Kampala, Uganda

## Abstract

Since 2012, the WHO recommends lifelong ART with TDF+FTC/3TC+EFV for all HIV-positive pregnant and breastfeeding women (Option B-plus). In this analysis we describe the proportion of early and late transmission in mothers with high retention in Kampala, Uganda. We included 700 pregnant women from January 2012 to August 2014 with a follow-up extended to August 2016; the median age was 31 years (IQR: 26–35), 36.3% in WHO stage 3/4; median CD4 count was 447 cells/*μ*L (IQR: 301–651) and 73.3% were already on ART for a median time of 28 (IQR: 10–57) months; 52% infants were male and median weight was 3.2 Kg (IQR: 2.5–3.5). Five hundred and sixty-five (80.7%) infants had at least one test for HIV; 22 (3.1%) infants died, all with unknown serostatus; 3 tested positive at week 6 and one additional at months 12 and 18. Two of the mothers of the 4 HIV-positive infants were ART-naïve at the time of pregnancy. We report very low documented HIV transmission comparable with those reported in clinical trials settings; however, demonstrating the efficacy of Option B-plus in terms of averted transmission in routine settings is challenging since high proportion of infants do not have documented HIV tests.

## 1. Introduction

Since 2012 the WHO recommends immediate start of lifelong ART as a combination of TDF+FTC/3TC+EFV for all HIV-positive pregnant and breastfeeding women, a practice known as Option B-plus, in order to reduce mother to child transmission (MTCT) [1]. In the PROMISE trial the estimates of HIV MTCT at ages of 6, 9, and 12 months were 0.3%, 0.5%, and 0.6% [2], well below the UNAIDS set target for MTCT transmission rated of less than 5% [3]. However, currently there is paucity of data to evaluate the true efficacy of this strategy in routine settings. Program data of exposed infants whose mothers were enrolled into Option B-plus and who tested for HIV infection from Kenya [4] and Malawi [5] show a transmission rate of 2.8% and 5.9%, respectively.

In this study we explored the feasibility of reporting the efficacy of Option B-plus in averting HIV transmission in a care setting with a high retention from enrolment to 18 months postpartum [6].

## 2. Methods

### 2.1. Study Setting and Population

The Infectious Diseases Institute (IDI), Makerere University, is an HIV centre of excellence [7] in Kampala, Uganda, with over 8,000 HIV-positive individuals receiving care. Since 2012 all pregnant women are seen in an integrated HIV-antenatal care clinic where they receive ART under Option B-Plus and they are educated about obstetric practices and breastfeeding options [6]. Postpartum, the women are instructed to attend the visits with their infants at the clinic Mother Baby Care Point. Infant feeding options are explained to the mother, with exclusive breastfeeding for the first 6 months followed by exclusive replacement feeding being the preferred option. Exposed infants receive daily nevirapine from birth until 6 weeks of age and are tested for HIV by DNA PCR at week 6 and month 12 using Roche COBAS AmpliPrep/COBAS TaqMan (CAP/CTM) HIV-1 assay, and at month 18 by serial rapid testing algorithm with Determine, STAT-PAK, and Uni-Gold to prompt early infant diagnosis.

The data is entered by the health care providers into a specialized electronic medical record for mother and baby pairs [8]. For this analysis we included women enrolled from January 2012 to August 2014 with a follow-up extended to August 2016, retained into care up to the end of the pregnancy and with a still birth. We extracted from the database baseline demographic characteristics, WHO stage, CD4 count, trimester of enrolment into the integrated HIV-antenatal care clinic, and ART status at the time of pregnancy. Viral loads were not routinely performed during the study period. Infants HIV-DNA PCR and antibody test results were traced in the clinic files and entered in the database. We described proportion of early transmission and late transmission.

### 2.2. Ethical Statement

This study was approved by the School of Medicine Research and Ethics Committee, Makerere University (Reference number 2009-120), and the Uganda National Council for Science and Technology; the investigators obtained waiver from verbal or written consent to analyse routinely collected data after stripping it of unique personal identifiers.

## 3. Results

Seven hundred women were included in the analysis, the median age was 31 years (IQR: 26–35), 534 (74.9%) joined the integrated HIV-antenatal care clinic during the 1st or 2nd trimester, and the parity was >2 for 329 (39.5%) women. Two hundred and fifty-four (36.3%) were in WHO stage 3 or 4; the median CD4 count was 447 (IQR: 301–651) cells/*μ*L; 33.5% had a CD4 count ≤ 350 cells/*μ*L, 33.5% between 351 and 500 cells/*μ*L and 42.6% > 500 cells/*μ*L. Most of the women (513, 73.3%) were already on ART for a median time of 28 months (IQR: 10–57) (Table 1). The majority (93.6%) were retained in care up to 18 months after giving birth, 3 (0.4%) died, 18 (2.6%) were lost to care, and 24 (3.4%) were transferred out to another facility.

Three hundred and sixty-four (52%) of the infants were female, and the median weight at birth was 3.2 Kg (IQR: 2.5–3.5). Five hundred and sixty-five (80.7%) infants had at least one test for HIV. We compared the characteristics of the mother whose infants received at least test with those whose infants were not tested, and we found that older mothers (median age 31 years, IQR: 26–35 versus 28, IQR: 24–34, *P* value = 0.03), those who were still retained 18 months after giving birth (98.4% versus 71.1%, *P* value < 0.0001), and those breastfeeding (87.7% versus 74.4%, *P* value < 0.0001) were more likely to have their infants tested for HIV.

Table 2 shows the numbers of infants tested and the serostatus of the babies at the different testing points. The proportion of infants tested was 80.3% at week 6, 69.5% at month 12, and 66.7% at month 18; 22 (3.1%) infants, all with unknown serostatus, died. Three of the infants tested positive at week 6 and one additional at months 12 and 18. Two of the mothers of the 4 infants who tested positive were ART-naïve at the time of pregnancy.

## 4. Conclusion

In this study we explored the feasibility of reporting the efficacy of Option B-plus in an HIV centre of excellence which provides integrated HIV-antenatal care and with high levels of postpartum retention achieved (93%).

We found very low documented HIV transmission comparable with those reported in clinical trials settings (PROMISE study, 13 sites in sub-Saharan Africa: 0.6% at month 12 [2]). However nearly one-fifth of the infants in our clinic were not tested for HIV.

At present there is limited information on the serostatus of HIV exposed infants whose mothers were enrolled in the Option B-Plus programs in sub-Saharan Africa due to poor documentation and high rates of lost to program, up to 68% [9], with a postpartum lost to follow-up 49% [10], implying that half of the infants are not tested for HIV. Given that the infants born of mother lost from care may be even at a higher risk of HIV transmission; it is likely that the current rates on HIV transmission from mother to child, which rely solely on infants that are brought back to the clinics to be tested, are underestimated [4, 5].

In our program we found a high level of retention (93.1%); however this was not mirrored by the number of infants tested at least once (80.7%) with the majority (71%) of the untested kids born of retained mothers; additionally infants were not consistently tested with 19%, 30%, and 33% of the infants of retained mothers not receiving HIV testing at week 6 and months 12 and 18, respectively. This was mainly attributed to the mothers not attending the clinic with their infants rather than lack of documentation. Staff working in the Mother Baby Care point reports that many women did not disclose their serostatus to their partners, making it difficult to justify going to the hospital with their babies. Other reasons for attending the visits alone were the burden of carrying the babies especially when coming from far using public transport; additionally, in some instances, the women separated from their partners after giving birth and the baby was living with the father.

Thus the results of our study demonstrate that, even in the context of high retention, close follow-up, integrated antenatal and mother-baby care, and good quality of data, it is not feasible to report on rates of vertical transmission using the conventional clinic based infant testing approach. Community based strategies including tracking and field testing may be necessary to demonstrate the true efficacy of Option B-plus in terms of averted HIV transmission to infants in routine settings, where mothers are lost to care or do not come back to the clinics with their babies.

In conclusion, tracking strategies and infant HIV testing in the field are needed to understand the true HIV vertical transmission rates and more importantly to early identify those in need to start antiretroviral treatment.

## Figures and Tables

**Table 1:** Baseline and follow-up characteristics of the 700 women enrolled in the HIV-antenatal integrated clinic and retained into care up to the end of the pregnancy.

Characteristics	Total *N* = 700 (100%)	Infant tested *N* = 565 (80.7%)	Infant not tested *N* = 135 (19.3%)	*P* value
Age (years), median (IQR)	31 (26–35)	31 (26–35)	28 (24–34)	0.03
Stage of pregnancy, *N* (%)				
1st trimester	233 (33.3)	187 (33.1)	46 (34.1)	0.87
2nd trimester	301 (43.0)	242 (42.8)	59 (43.7)	
3rd trimester	153 (21.9)	125 (22.1)	28 (20.7)	
On delivery	3 (0.4)	2 (0.4)	1 (0.7)	
After birth	10 (1.4)	9 (1.6)	1 (0.7)	
WHO clinical stage 3/4, *N* (%)	254 (36.3)	204 (36.1)	50 (37.0)	0.84
Parity > 2, *N* (%)	329 (39.5)	239 (43.6)	51 (38.1)	0.24
CD4 cells/*µ*L, median (IQR) *N* (%)	447 (301–651)	459 (307–658)	529 (277–593)	0.14
≤350	234 (33.5)	186 (33.0)	48 (35.6)	0.09
351–500	167 (23.9)	127 (22.6)	40 (29.6)	
>500	297 (42.6)	250 (44.4)	47 (34.8)	
Already on ART, *N* (%)	513 (73.3)	418 (74.0)	95 (70.4)	0.39
Feeding option, breastfeeding	590 (85.1%)	491 (87.7%)	99 (74%)	<0.001
Follow up status, *N* (%)				
Retained	655 (93.6%)	558 (98.8)	97 (71.8)	<0.001
Dead	3 (0.4%)	0 (0.0)	3 (2.2)	
Lost	18 (2.6%)	4 (0.7)	14 (10.4)	
Transferred out	24 (3.4%)	3 (0.5)	21 (15.6)	

*N* = number; ART: antiretroviral treatment.

**Table 2:** Number and proportion of mother retained in care and results infants tested for HIV at 6 weeks and 12 and 18 months after delivery.

Time of HIV test	Mothers retained	*N* (%) positive	*N* (%) negative	*N* (%) unknown
6 weeks	700 (100%)	3 (0.4)	562 (80.3)	135 (19.3)
12 months	675 (96.4%)	4 (0.6)	469 (69.5)	202 (29.9)
18 months	655 (93.6%)	4 (0.6)	437 (66.7)	216 (32.7)
